# Supplementary material for: Pyruvate Dehydrogenase Kinase 1 inhibition mediated oxidative phosphorylation enhancement in cartilage promotes osteoarthritis progression
Source: BMC Musculoskelet Disord. 2023 Jul 20;24:597. doi: 10.1186/s12891-023-06585-6 (PMC10357736; doi:10.1186/s12891-023-06585-6)
Supplement: Supplementary file 1 — Additional file 1. [file 12891_2023_6585_MOESM1_ESM.zip › KEGG.Gsea.1653622432464/KEGG_STEROID_BIOSYNTHESIS.html]

Details for gene set KEGG\_STEROID\_BIOSYNTHESIS[GSEA]

|  || Dataset | 55457\_collapsed\_to\_symbols.cls.cls #OA\_versus\_normal.cls.cls #OA\_versus\_normal\_repos |
| Phenotype | cls.cls#OA\_versus\_normal\_repos |
| Upregulated in class | OA |
| GeneSet | KEGG\_STEROID\_BIOSYNTHESIS |
| Enrichment Score (ES) | 0.17212583 |
| Normalized Enrichment Score (NES) | 0.54229236 |
| Nominal p-value | 0.97820824 |
| FDR q-value | 0.9951095 |
| FWER p-Value | 1.0 |
Table: GSEA Results Summary

  

Fig 1: Enrichment plot: KEGG\_STEROID\_BIOSYNTHESIS      
 Profile of the Running ES Score & Positions of GeneSet Members on the Rank Ordered List

  

| SYMBOL | TITLE | RANK IN GENE LIST | RANK METRIC SCORE | RUNNING ES | CORE ENRICHMENT || 1 | SC5D | sterol-C5-desaturase [Source:HGNC Symbol;Acc:HGNC:10547] | 319 | 0.738 | 0.1360 | Yes |
| 2 | LIPA | "lipase A, lysosomal acid type [Source:HGNC Symbol;Acc:HGNC:6617]" | 1836 | 0.380 | 0.1011 | Yes |
| 3 | FDFT1 | farnesyl-diphosphate farnesyltransferase 1 [Source:HGNC Symbol;Acc:HGNC:3629] | 2538 | 0.299 | 0.1117 | Yes |
| 4 | DHCR24 | 24-dehydrocholesterol reductase [Source:HGNC Symbol;Acc:HGNC:2859] | 3126 | 0.237 | 0.1178 | Yes |
| 5 | MSMO1 | methylsterol monooxygenase 1 [Source:HGNC Symbol;Acc:HGNC:10545] | 3146 | 0.235 | 0.1675 | Yes |
| 6 | EBP | EBP cholestenol delta-isomerase [Source:HGNC Symbol;Acc:HGNC:3133] | 3848 | 0.171 | 0.1503 | Yes |
| 7 | SQLE | squalene epoxidase [Source:HGNC Symbol;Acc:HGNC:11279] | 4005 | 0.156 | 0.1721 | Yes |
| 8 | TM7SF2 | transmembrane 7 superfamily member 2 [Source:HGNC Symbol;Acc:HGNC:11863] | 4410 | 0.120 | 0.1670 | No |
| 9 | LSS | lanosterol synthase [Source:HGNC Symbol;Acc:HGNC:6708] | 5182 | 0.058 | 0.1197 | No |
| 10 | SOAT2 | sterol O-acyltransferase 2 [Source:HGNC Symbol;Acc:HGNC:11178] | 6750 | -0.059 | 0.0109 | No |
| 11 | SOAT1 | sterol O-acyltransferase 1 [Source:HGNC Symbol;Acc:HGNC:11177] | 7555 | -0.124 | -0.0245 | No |
| 12 | CEL | carboxyl ester lipase [Source:HGNC Symbol;Acc:HGNC:1848] | 8375 | -0.184 | -0.0481 | No |
| 13 | DHCR7 | 7-dehydrocholesterol reductase [Source:HGNC Symbol;Acc:HGNC:2860] | 8626 | -0.203 | -0.0234 | No |
| 14 | CYP27B1 | cytochrome P450 family 27 subfamily B member 1 [Source:HGNC Symbol;Acc:HGNC:2606] | 9582 | -0.282 | -0.0361 | No |
| 15 | CYP51A1 | cytochrome P450 family 51 subfamily A member 1 [Source:HGNC Symbol;Acc:HGNC:2649] | 9598 | -0.283 | 0.0244 | No |
| 16 | NSDHL | NAD(P) dependent steroid dehydrogenase-like [Source:HGNC Symbol;Acc:HGNC:13398] | 11670 | -0.527 | -0.0215 | No |
| 17 | HSD17B7 | hydroxysteroid 17-beta dehydrogenase 7 [Source:HGNC Symbol;Acc:HGNC:5215] | 11723 | -0.537 | 0.0914 | No |
Table: GSEA details [plain text format]

  

Fig 2: KEGG\_STEROID\_BIOSYNTHESIS      
 Blue-Pink O' Gram in the Space of the Analyzed GeneSet

  

Fig 3: KEGG\_STEROID\_BIOSYNTHESIS: Random ES distribution      
 Gene set null distribution of ES for **KEGG\_STEROID\_BIOSYNTHESIS**
